# Real-World Pharmacokinetics, Effectiveness, and Safety of Atezolizumab in Patients With Unresectable Advanced or Recurrent NSCLC: An Exploratory Study of J-TAIL

**DOI:** 10.1016/j.jtocrr.2024.100683

**Published:** 2024-05-16

**Authors:** Shigehiro Yagishita, Yasushi Goto, Makoto Nishio, Hiroaki Akamatsu, Hidetoshi Hayashi, Satoru Miura, Koji Tamada, Hiroshi Kagamu, Akinobu Hamada, Mayu Ohuchi, Akihiko Gemma, Ichiro Yoshino, Toshihiro Misumi, Akito Hata, Satoshi Hara, Takashi Kijima, Fujita Masaki, Shunichiro Iwasawa, Shintaro Nakagawa, Masahiro Tatsuno, Tetsuya Mitsudomi

**Affiliations:** a Division of Molecular Pharmacology, National Cancer Center Research Institute, Tokyo, Japan; b Department of Thoracic Oncology, National Cancer Center Hospital, Tokyo, Japan; c Department of Thoracic Medical Oncology, The Cancer Institute Hospital of Japanese Foundation for Cancer Research, Tokyo, Japan; d Internal Medicine III, Wakayama Medical University, Wakayama, Japan; e Department of Medical Oncology, Faculty of Medicine, Kindai University, Osaka, Japan; f Department of Internal Medicine, Niigata Cancer Center Hospital, Niigata, Japan; g Department of Immunology, Yamaguchi University Graduate School of Medicine, Yamaguchi, Japan; h Department of Respiratory Medicine, International Medical Center, Saitama Medical University, Saitama, Japan; i Department of Pharmacology and Therapeutics, Fundamental Innovative Oncology Core, National Cancer Center Research Institute, Tokyo, Japan; j Department of Pulmonary Medicine and Oncology, Nippon Medical School, Graduate School of Medicine, Tokyo, Japan; k Department of General Thoracic Surgery, Chiba University Graduate School of Medicine, Chiba, Japan; l Department of Thoracic Surgery, International University of Health and Welfare Narita Hospital, Narita, Japan; m Department of Biostatistics, Yokohama City University School of Medicine, Kanagawa, Japan; n Department of Data Science, National Cancer Center Hospital East, Chiba, Japan; o Department of Thoracic Oncology, Kobe Minimally Invasive Cancer Center, Hyogo, Japan; p Department of Respiratory Medicine, Itami City Hospital, Hyogo, Japan; q Department of Respiratory Medicine and Hematology, Hyogo Medical University, Nishinomiya, Hyogo, Japan; r Department of Respiratory Medicine, Faculty of Medicine, Fukuoka University, Fukuoka, Japan; s Chugai Pharmaceutical Co., Ltd., Tokyo, Japan; t Kindai Hospital Global Research Alliance Center and Thoracic Surgery, Kindai University, Osaka, Japan

**Keywords:** Atezolizumab, Effectiveness, Non–small cell lung cancer, Pharmacokinetics, Real-world

## Abstract

**Introduction:**

This study validated real-world pharmacokinetic (PK) data using an established population PK (PopPK) model for atezolizumab in Japanese patients with NSCLC and explored the relationship between PK parameters, effectiveness, and adverse events (AEs) for the 1200 mg once every three weeks regimen.

**Methods:**

A subgroup of 262 of 1039 patients from J-TAIL consented to this exploratory research for PK evaluation of atezolizumab monotherapy for unresectable advanced/recurrent NSCLC (August 2018 to October 2019; 197 institutions). We evaluated plasma concentrations before the start of the third cycle of atezolizumab infusion classified into quartiles 1 to 4, their association with effectiveness, and the association between atezolizumab maximum plasma concentrations (C_max_) calculated using the existing PopPK model and AEs of special interest (AESIs).

**Results:**

Overall, 175 of 262 patients were included; baseline characteristics were similar to those of patients enrolled in J-TAIL (Eastern Cooperative Oncology Group performance status ≥ 2, 12.0%; age ≥ 75 y, 28.9%; atezolizumab as more than or equal to third-line treatment, 57.5%). Atezolizumab plasma concentrations were similar to previously reported data among Japanese/non-Japanese patients. The overall survival was significantly shorter in patients with lower atezolizumab plasma concentrations in Q1 versus Q2 to Q4, although progression-free survival remained the same. The PK data adequately fit the PopPK model, with the frequency of AESIs increasing as the calculated C_max_ at cycle 1 increased.

**Conclusions:**

In real-world Japanese patients with unresectable advanced/recurrent NSCLC, PKs were similar to previous reports. Certain patient populations had shorter overall survival, and atezolizumab plasma concentrations in cycle 3 were lower in this population. Elevated C_max_ at cycle 1 may be associated with an increased frequency of AESIs.

## Introduction

The 5-year survival rate for patients with advanced NSCLC treated with conventional cytotoxic chemotherapy was less than 5%.^1^ Various driver gene alterations have been identified in NSCLC, and molecularly targeted therapies have provided considerable survival gains. Nevertheless, treatments for patients without driver gene alterations remain limited. In this context, immune checkpoint inhibitors (ICIs) have become a treatment option for patients with advanced NSCLC, having clinically meaningful survival benefit, durable responses, and favorable safety profiles compared with chemotherapy.^2^, ^3^, ^4^, ^5^, ^6^ Thus, ICIs have become a key drug for patients with advanced NSCLC.

Among ICIs, atezolizumab is a recombinant humanized monoclonal antibody of the immunoglobulin G (IgG) 1 subclass against human programmed death-ligand 1 expressed in tumor or immune cells. During clinical development of atezolizumab, a dose-escalation study assessing the dosing regimen of 0.3 to 20 mg/kg once every three weeks found a dose-dependent increase in exposure without an increase in the frequency of toxicity.^7^ Consequently, a shift to a fixed-dose regimen was considered, which provided the advantages of eliminating dose adjustment, eliminating residual drug disposal during dose adjustment, and facilitating simplicity of prescribing. On the basis of the population pharmacokinetic (PopPK) model, atezolizumab 1200 mg/body once every three weeks (equivalent to 15 mg/kg exposure) was considered the recommended dose for transition to the phase II trial. Subsequently, the multicenter, open-label, single-arm phase II BIRCH and the international phase III OAK studies revealed that 1200 mg/body once every three weeks dosing was able to achieve similar atezolizumab plasma concentrations among Japanese and non-Japanese patients.^5^^,^^8^^,^^9^

The characteristics of patients enrolled in clinical trials often differ from those of patients encountered in the real world due to the strict eligibility criteria in clinical trials. In the real world, atezolizumab can be administered to a diverse patient population, including elderly patients or those with poor performance status, various organ dysfunctions, and cachexia. There are concerns that the pharmacokinetics (PK) of atezolizumab may be altered in such patient populations due to in vivo inflammation and metabolic abnormalities, which may ultimately affect treatment efficacy and the frequency of immune-related adverse events (irAEs) and highlight differences in outcomes to those observed in clinical studies.^10^^,^^11^ Furthermore, PK data of atezolizumab in the Japanese population are limited, especially in the real-world setting.^12^

On the basis of this background, we decided to perform a PK analysis of atezolizumab in a companion study to the J-TAIL study, which evaluated the efficacy and safety of atezolizumab monotherapy in Japanese patients with unresectable advanced/recurrent NSCLC.^13^ The objectives of this study were to obtain real-world PK data of atezolizumab in Japanese patients with NSCLC, validate the PK data using established PopPK models,^12^ and explore the relationship between PK parameters and the efficacy and safety of the atezolizumab 1200 mg once every three weeks regimen in real-world clinical practice.^12^

## Materials and Methods

### Study Design

J-TAIL was a prospective, single-arm, noninterventional, nonblinded observational study that aimed to evaluate the safety and efficacy of atezolizumab monotherapy in Japanese patients with unresectable advanced/recurrent NSCLC.^13^ A total of 1039 consecutive patients scheduled to receive atezolizumab monotherapy were enrolled from August 15, 2018, to October 16, 2019, at 197 institutions in Japan. A subgroup of 262 patients (73 institutions) consented to this exploratory ancillary research of J-TAIL.

This study was conducted in accordance with the Declaration of Helsinki, Ethical Guidelines for Medical and Health Research Involving Human Subjects, and the International Council for Harmonisation guidelines for Good Clinical Practice. All patients provided written informed consent for study participation. This study is registered at UMIN-CTR under the identifier number UMIN000035567.

### Patients and Treatment

Detailed eligibility criteria for J-TAIL have been previously published.^13^ Briefly, patients above or equal to 20 years of age who were previously treated with systemic therapy for unresectable advanced/recurrent NSCLC and scheduled to receive atezolizumab monotherapy were enrolled. Patients were excluded if they were considered unsuitable for enrollment at the investigator’s discretion.

Eligible patients received atezolizumab monotherapy according to the instructions in the latest package insert.^8^ Dose interruptions or withdrawals of atezolizumab were based on the physician’s discretion with guidance from the atezolizumab package insert and the Guidelines for the Promotion of Optimal Use.^14^

### End Points

The primary end point of this study was to evaluate the PK of atezolizumab monotherapy in Japanese patients with unresectable advanced/recurrent NSCLC in real-world clinical practice. The PK data were validated using an established PopPK model for atezolizumab described subsequently.^12^ Secondary end points included correlation analysis of atezolizumab PK with efficacy and AEs of special interest (AESIs) and all grade greater than or equal to III AEs. Among the events reported as irAE by the investigators, the following events were defined as AESIs in this study: encephalitis, meningitis, autoimmune hemolytic anemia, adrenal gland failure, adrenal insufficiency, hyperthyroidism, hypopituitarism, hypothyroidism, secondary adrenocortical insufficiency, type 1 diabetes mellitus, severe type 1 diabetes mellitus, myasthenia gravis, myocarditis, interstitial lung disease, pneumonia, colitis, diarrhea, autoimmune hepatitis, abnormal liver function, liver damage, immune hepatitis, rash, myositis, tubulointerstitial nephritis, and reactions associated with injection.

Disease progression and treatment response were assessed by the investigators according to Response Evaluation Criteria in Solid Tumors version 1.1,^15^ with confirmatory measurements. As J-TAIL was an observational study, the timing and method of these assessments were determined at the discretion of the investigators and institutions.

### PK Analysis

Blood samples for PK analysis were collected before initiating atezolizumab infusion in cycle 1 (C1, henceforth called “baseline”) and cycle 3 (trough concentration [C_trough_] of C2; henceforth called “C2_trough_”) and at the onset of an irAE. Samples collected more than seven days before the first infusion of atezolizumab and more than three days before the third infusion were excluded from the analysis to ascribe the permissible range for measurement. Atezolizumab plasma concentrations were measured using liquid chromatography–mass spectrometry (LC-MS) equipped with a triple quadrupole mass analyzer (Nexera X2 and LC-MS-8050, Shimadzu, Kyoto, Japan) as previously described, with some modifications.^10^ Briefly, atezolizumab and rituximab (as an internal standard) in blood samples were prepared using the “nano-surface and molecular-orientation limited proteolysis” method.^16^ Atezolizumab and rituximab were digested and extracted to obtain the signature peptides RHWPGGFDYWGQGTLVTVSSASTK and GLEWIGAIYPGNGDTSYNQK, respectively. The calibration curve range of atezolizumab was 5 to 600 μg/mL, and quantitative accuracy, reproducibility, and stability were confirmed using triplicate experimental replicates (Supplementary Table 1). The selectivity, lower limit of quantification, calibration curve, carryover, accuracy, and stability met the requirements of bioanalytical guidance (Supplementary Tables 2–4).^17^

### PopPK Analysis

#### PopPK Model

We used the initial PopPK model developed by Stroh et al.^12^ using data from two phase I studies where a linear PK of atezolizumab was found over a dose range of 1 to 20 mg/kg, including the 1200 mg labeled dose. The clearance (CL), volume of distribution, and terminal half-life of atezolizumab were 0.2 L/d, 6.9 L, and 27 days, respectively. Covariates included body weight, albumin (Alb), tumor volume, immunogenicity (treatment-emergent anti-drug antibody) on CL and Alb, body weight, and sex effect on volume of distribution. This model was used for external validation using data from the J-TAIL study.

Data inputs for the model were taken from analysis-ready source data sets of J-TAIL and the assessable PK population of this ancillary study. Data assembly was performed using R software (version 4.1.1), and quality control checks were performed using Certara’s standard operating procedures.

The assessable patients for PopPK analysis (n = 175) had to have received at least one dose of atezolizumab with relevant dosing information available before each measurable concentration, at least one measurable postdose concentration with a corresponding sample time, and no protocol violation or event that could confound the interpretation of the PK analyses. To explore the relationship between PK exposure and AESIs, patients were required to be part of both the PopPK data set of this study and the safety analysis data set of J-TAIL.

For covariates that were included in the previous PopPK model^12^ but missing for less than or equal to 10% of patients in the current study data set (e.g., immunogenicity), values were imputed to the reference category for categorical covariates or the previously reported median value for continuous covariates. Covariates missing for greater than 10% of patients were excluded. No excluded outlier data were flagged in the PK analysis data set of this study. The analyses were carried out according to the United States Guidance for Industry: Population Pharmacokinetics,^17^ Japan Pharmaceuticals and Medical Devices Agency guideline,^18^ and the European Union Guidance on Reporting the Results of Population Pharmacokinetic Analyses.^19^

Atezolizumab plasma concentration–time data were analyzed using nonlinear mixed-effects modeling software (NONMEM, version 7.4.3; ICON, Hanover, MD). Analyses were performed using the first-order conditional estimation method with interaction. R software (version 4.2.0) was used for postprocessing of all analysis outputs, goodness-of-fit, and descriptive statistics.

#### Visual Predictive Checks

For visual predictive checks, the PopPK model^12^ was used to validate atezolizumab plasma concentrations in this study without any re-estimations (by setting “MAXEVAL” to 0) and imputing missing covariates to the reference value. A prediction-corrected visual predictive check and individual time–concentration profiles were then used to address whether the model adequately described the data in this study. The external validation was considered successful, given the consistency between model predictions and observed atezolizumab plasma concentrations from this study. The goodness-of-fit of the model was evaluated by (1) visual predictive checks and (2) time–concentrations profiles from 0 to 80 days using the estimated individual PK parameters, post hoc, to obtain the full profiles of patients and evaluate model fitness.

A total of 1000 simulations based on the entire study population of J-TAIL were performed using the observed covariates for each patient, final model parameter estimates, estimated patient-specific random effects, and residual errors. The observed data in this study were overlaid and visually compared with the confidence interval (CI) for the time course of the predicted median and spread of concentrations (i.e., fifth to 95th percentile).^20^^,^^21^ In addition, time–concentration profiles were sampled for 80 days, with sampling at every hour. Dosing was followed based on the source data. Time–concentration profiles were calculated under the simulation block of NONMEM with 100 subproblems.

#### PopPK Model Simulations

Post hoc empirical Bayes estimates from the PopPK model were used to simulate concentration–time profiles for each patient in this study. Simulations were performed using the RxODE package (R version 4.2.0) to predict the derived exposure metrics (maximum concentration [C_max_] and area under the curve [AUC] in C1) after the atezolizumab 1200 mg once every three weeks regimen. Summary statistics of the derived exposure metrics (mean, standard deviation, and coefficient of variation [CV%]) are tabulated (Supplementary Table 5).

### Statistical Analyses

Descriptive statistics were used to summarize the baseline characteristics of patients using median (interquartile range or range) for continuous variables and n (%) for categorical variables. Single and multiple regression analyses were performed with atezolizumab plasma concentration as the objective variable. Multiple regression analyses were performed using the linear regression model, with plasma concentration as the objective variable and sex, body surface area (BSA), histologic subtype, smoking history, tumor volume, Alb, C-reactive protein (CRP), and atezolizumab treatment line as explanatory variables. The proportion of patients with complete response or partial response was calculated for objective response rate. For disease control rate (DCR), the proportion of patients with complete response, partial response, or stable disease maintained for more than 24 weeks was calculated; a normal approximation was used to calculate the 95% CIs. The Kaplan-Meier (KM) method was used to estimate overall survival (OS) and progression-free survival (PFS). The hazard ratio (HR) was estimated using stratified Cox regression analysis to compare the groups. Stratification factors were sex, age, Eastern Cooperative Oncology Group performance status (ECOG PS), histology, programmed death-ligand 1 tumor proportion score, stage, and atezolizumab plasma concentration. The association between C2_trough_ and OS was analyzed using a Cox proportional hazards model, with additional explanatory variables extracted based on the multivariate analysis results of the OAK study for OS.^5^ No imputation method was used for missing data. Logistic regression models were developed to describe the relationships between exposure metrics (C_max_ and AUC in C1) and individual occurrence of AESIs. In addition, each patient was categorized into whether a prespecified any-grade and grade greater than III AESI occurred or not. The relationship between exposure quartiles (quartiles of C_max_ and AUC in C1) and probability of AESI incidence was evaluated using logistic regression analysis. The significance level for the test was set at 5%, not considering multiplicity for exploratory purpose. All statistical analyses were conducted using SAS version 9.4 (SAS Institute Inc., Cary, NC).

## Results

### Patient Disposition and Baseline Characteristics

A total of 262 patients from the J-TAIL study consented to participate in this ancillary research; among them, 175 patients were included in the final analysis after excluding 87 patients (78 patients did not provide blood samples for C2_trough_ and nine patients had their samples collected more than 7 d before the first dose of atezolizumab; Supplementary Fig. 1). In the 175 patients analyzed, the median number of cycles of atezolizumab administration was four (minimum 2, maximum 34), with 16 patients (9.1%) receiving two cycles, 49 patients (28.0%) receiving three cycles, and 110 patients (62.9%) receiving four or more cycles.

The baseline characteristics of this subgroup of patients were generally similar to those of the overall patients enrolled in the J-TAIL study^13^; 28.9% versus 29.1% were above or equal to 75 years of age, 57.5% versus 55.4% were receiving atezolizumab as third-line and later treatment, and 71.8% versus 66.3% were male, which is anticipated in a real-world setting (Table 1). Furthermore, 6.3% of patients had an ECOG PS of greater than or equal to 2, which was higher in the J-TAIL study, at 12.0%.

**Table 1:** Patient Demographics and Clinical Characteristics

Characteristics, n (%)	Overall N = 175	Cycle 3 Atezolizumab Concentration		*p* Value^a^
		Q1 N = 43	Q2–Q4 N = 132	Q1 vs. Q2–Q4
Sex				< 0.001
Male	116 (66.3)	39 (90.7)	77 (58.3)	
Female	59 (33.7)	4 (9.3)	55 (41.7)	
Age, y				
Median age (range)	71 (41–90)	72 (41–86)	70 (42–90)	0.1831
≥ 75	51 (29.1)	16 (37.2)	35 (26.5)	
Height (cm), median [IQR]	163.1 [156.0–168.0]	165.0 [161.0–169.1]	162.4 [154.7–167.8]	0.0122
Weight (kg), median [IQR]	58.6 [49.4–66.0]	61.0 [52.2–66.3]	57.6 [48.7–65.9]	0.1384
BSA^b^ (m^2^), median [IQR]	1.63 [1.48–1.73]	1.66 [1.53–1.73]	1.62 [1.45–1.73]	0.0464
BMI (kg/m^2^), median [IQR]	21.9 [20.0–24.4]	22.0 [19.7–24.4]	21.6 [20.0–33.3]	0.1600
Histology				0.0022
Squamous	37 (21.1)	17 (39.5)	20 (15.2)	
Nonsquamous	135 (77.1)	25 (58.1)	110 (83.3)	
Other	3 (1.7)	1 (2.3)	2 (1.5)	
ECOG PS				0.3712
0	68 (38.9)	13 (30.2)	55 (41.7)	
1	96 (54.9)	26 (60.5)	70 (53.0)	
2	10 (5.7)	4 (9.3)	6 (4.5)	
3	1 (0.6)	0 (0.0)	1 (0.8)	
4	0 (0.0)	0 (0.0)	0 (0.0)	
Smoking history	127 (72.6)	38 (88.4)	89 (67.4)	0.0097
Primary tumor surgery	58 (33.1)	12 (27.9)	46 (34.8)	0.5910
Metastases				
Brain	33 (18.9)	8 (18.6)	25 (18.9)	1
Bone	48 (27.4)	13 (30.2)	35 (26.5)	0.6949
Adrenal	7 (4.0)	5 (11.6)	2 (1.5)	0.0104
Liver	14 (8.0)	2 (4.7)	12 (9.1)	0.5220
Kidney	1 (0.6)	1 (2.3)	0 (0.0)	0.2457
Other^c^	141 (80.6)	35 (81.4)	106 (80.3)	1
Stage				0.5744
IIIA	7 (4.0)	1 (2.3)	6 (4.5)	
IIIB	14 (8.0)	3 (7.0)	11 (8.3)	
IIIC	1 (0.6)	0 (0.0)	1 (0.8)	
IVA	41 (23.4)	14 (32.6)	27 (20.5)	
IVB	48 (27.4)	10 (23.3)	38 (28.8)	
Postsurgery recurrence	47 (26.9)	9 (20.9)	38 (28.8)	
Postchemoradiation therapy recurrence	17 (9.7)	6 (14.0)	11 (8.3)	
Treatment line of atezolizumab				0.3328
2	78 (44.6)	23 (53.5)	55 (41.7)	
3	26 (14.9)	4 (9.3)	22 (16.7)	
≥ 4	71 (40.6)	16 (37.2)	55 (41.7)	
PD-L1				
22C3				
n	138	38	100	0.4521
TPS ≥ 50%	29 (21.0)	10 (26.3)	19 (19.0)	
TPS 1%–49%	47 (34.1)	14 (36.8)	33 (33.0)	
TPS < 1%	62 (44.9)	14 (36.8)	48 (48.0)	
Tumor volume (mm), median [IQR]	43.9 [33.0–61.4]	52.0 [40.0–69.0]	41.5 [31.4–56.8]	0.1088
Alb (g/dL), median [IQR]	3.9 [3.6–4.1]	3.6 [3.3–3.9]	3.9 [3.7–4.2]	0.0001
CRP (mg/dL), median [IQR]	0.27 [0.11–0.19]	0.9 [0.3–2.4]	0.2 [0.1–0.7]	0.0065

Alb, albumin; BMI, body mass index; BSA, body surface area; CRP, C-reactive protein; ECOG PS, Eastern Cooperative Oncology Group performance status; IQR, interquartile range; PD-L1, programmed death-ligand 1; Q, quartile; TPS, tumor proportion score.

a Chi-square test or Wilcoxon ranked sum test.

b Du Bois formula.

c “Other” includes patients with pleural effusion.

### PK of Atezolizumab

Atezolizumab plasma concentrations at C2_trough_ were similar to those observed in patients with squamous or nonsquamous NSCLC in the OAK study^5^ (Supplementary Fig. 2). Patient characteristics categorized into quartiles of C2_trough_ had a significant difference in the proportion of patients by sex, BSA, histology, smoking history, presence of adrenal metastasis, Alb, and CRP between the lowest quartile (Q1) and other quartiles (Q2–Q4; Table 1). The proportion of patients with ECOG PS greater than or equal to 2 was not markedly different between the two groups, although the frequency was slightly higher among patients in Q1 versus Q2 to Q4 (9.3% versus 5.3%).

**Figure 1:**
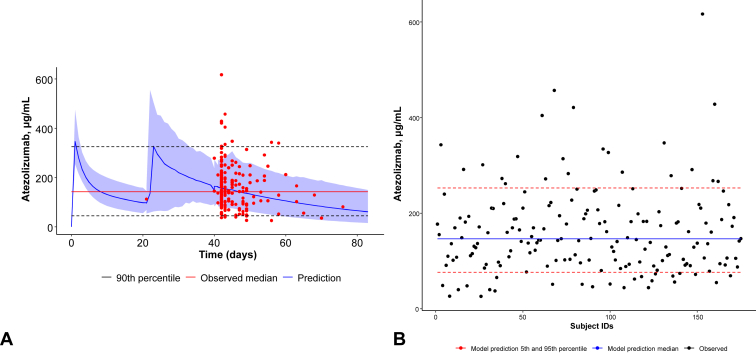
Simulated time–concentration profiles based on individual PK parameters: PopPK model of atezolizumab constructed by (*A*) Stroh et al.^12^ and (*B*) comparison between the observed concentrations and model predictions (model fit). PK, pharmacokinetic; PopPK, population pharmacokinetic.

Using the PopPK model of atezolizumab constructed by Stroh et al.,^12^ we confirmed the PK of the analysis population (Supplementary Table 5) in this study and its conformity to the previously reported data (Fig. 1*A*). The PK analysis data set included 185 PK observations from 175 patients. In the data set, 165 patients had one PK sample and 10 patients had two PK samples. The results confirmed that the real-world data of Japanese patients treated with atezolizumab obtained in this study adequately fit Stroh’s model; most observations were within the fifth and 90th percentiles of the model predictions (Fig. 1*B*).

### Correlation Analysis of Atezolizumab PK With Effectiveness

**Figure 2:**
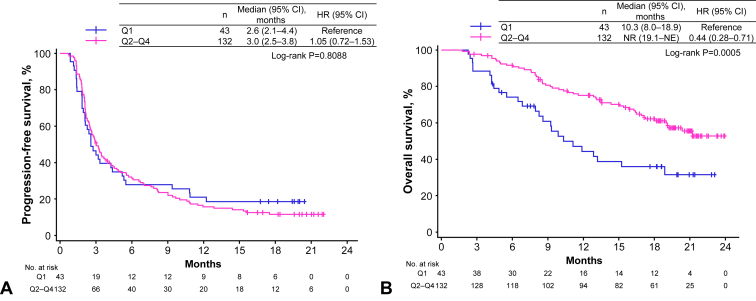
KM analysis of (*A*) PFS compared between Q1 versus Q2 to Q4 as a cumulative group and (*B*) OS compared between Q1 versus Q2 to Q4 as a cumulative group. CI, confidence interval; HR, hazard ratio; KM, Kaplan-Meier; NE, not evaluable; NR, not reached; OS, overall survival; PFS, progression-free survival; Q, quartile.

No clear correlation was observed between C2_trough_ and response rate (Table 2). Using KM analysis, there was also no significant difference in median PFS between Q1 (2.6 mo; 95% CI: 2.1–4.4) and Q2 to Q4 (3.0 mo; 95% CI: 2.5–3.8) for C2_trough_ (HR, 1.05; 95% CI: 0.72–1.53; log-rank *p* = 0.8088; Fig. 2*A*). PFS data comparing C2_trough_ of Q1 with those of Q2, Q3, and Q4 are found in Supplementary Fig. 3*A*.

**Table 2:** Association Between Response and Atezolizumab Concentration at Cycle 3

Response, n (%)	Cycle 3 Atezolizumab Concentration		*p* Value^a^
	Q1 N = 43	Q2–Q4 N = 132	
BOR			
CR	1 (2.3)	2 (1.5)	0.4853
PR	8 (18.6)	13 (9.8)	
SD	15 (34.9)	58 (43.9)	
PD	18 (41.9)	54 (40.9)	
NE	1 (2.3)	5 (3.8)	
ORR			
n (%)	9 (20.9)	15 (11.4)	0.1286
95% CI	[8.8–33.1]	[5.9–16.8]	
DCR			
n (%)	13 (30.2)	24 (18.2)	0.1307
95% CI	[16.5–44.0]	[11.6–24.8]	

BOR, best overall response; CI, confidence interval; CR, complete response; DCR, disease control rate; NE, not evaluable; ORR, objective response rate; PD, progressive disease; PR, partial response; Q, quartile; SD, stable disease.

a Chi-square test.

Median OS was significantly longer in patients in Q2 to Q4 (not reached; 95% CI: 19.1–not evaluable [NE]; HR, 0.44; 95% CI: 0.28–0.71; *p* = 0.0005) than in Q1 (10.3 mo; 95% CI: 8.0–18.9; Fig. 2*B*). HRs versus Q1 were as follows: Q2 (0.52; 95% CI: 0.28–0.93), Q3 (0.30; 95% CI: 0.15–0.59), and Q4 (0.53; 95% CI: 0.30–0.95), with a significant difference in OS between the four groups of Q1, Q2, Q3, and Q4 (log-rank *p* = 0.0023 for all four groups; Supplementary Fig. 3*B*).

The results of multivariate analysis revealed that poor ECOG PS and lower atezolizumab plasma concentrations were independent factors for OS and were significantly associated with poor prognosis (Table 3).

**Table 3:** Association Between OS and Atezolizumab Concentration at Cycle 3

Characteristics	Univariate		Multivariate	
	HR (95% CI)	*p* Value	HR (95% CI)	*p* Value
Sex				
Male	(Reference)	0.6785	(Reference)	0.3625
Female	0.91 (0.57–1.45)		1.39 (0.68–2.83)	
Age				
<75 y	(Reference)	0.0261	(Reference)	0.5661
≥75 y	1.68 (1.06–2.66)		1.19 (0.66–2.14)	
ECOG PS				
0–1	(Reference)	<0.0001	(Reference)	0.0056
≥2	4.33 (2.22–8.47)		4.15 (1.52–11.35)	
Histology				
Squamous	(Reference)	0.0883	(Reference)	0.4690
Nonsquamous/other	0.65 (0.39–1.07)		0.79 (0.42–1.49)	
PD-L1 TPS				
≥50%	(Reference)	0.7743	(Reference)	0.8287
1%–49%	0.94 (0.48–1.86)		1.00 (0.48–2.10)	
<1%	0.81 (0.41–1.57)		0.84 (0.41–1.72)	
Stage				
III	(Reference)	0.4352		
IV	1.50 (0.70–3.19)			
Postsurgery/postchemoradiation therapy recurrence	1.67 (0.77–3.66)			
Atezolizumab treatment line				
Second-line	1.23 (0.79–1.92)	0.3578	(Reference)	0.5851
Third-line or later			1.17 (0.67–2.05)	
Atezolizumab concentration				
Q1	(Reference)	0.0008	(Reference)	0.0030
Q2–Q4	0.44 (0.28–0.71)		0.37 (0.19–0.71)	
Height				
<Median (163.1 cm)	(Reference)	0.5538		
≥Median (163.1 cm)	0.87 (0.56–1.36)			
Smoking history				
No	(Reference)	0.5550	(Reference)	0.1714
Yes	0.86 (0.53–1.40)		0.59 (0.28–1.25)	
Adrenal metastasis				
Without	(Reference)	0.6584		
With	0.73 (0.18–2.97)			
Tumor volume				
<Median (43.9 mm)	(Reference)	0.2354		
≥Median (43.9 mm)	1.35 (0.82–2.23)			
Alb				
<Median (3.9 g/dL)	(Reference)	0.1873	(Reference)	0.6017
≥Median (3.9 g/dL)	0.74 (0.47–1.16)		0.86 (0.49–1.51)	
CRP				
<Median (0.27 mg/dL)	(Reference)	0.1822		
≥Median (0.27 mg/dL)	1.35 (0.87–2.12)			

Alb, albumin; CI, confidence interval; CRP, C-reactive protein; ECOG PS, Eastern Cooperative Oncology Group performance status; HR, hazard ratio; OS, overall survival; PD-L1, programmed death-ligand 1; Q, quartile; TPS, tumor proportion score.

### Correlation Analysis of Atezolizumab PK With AESIs and All Grade Greater Than or Equal to III AEs

**Figure 3:**
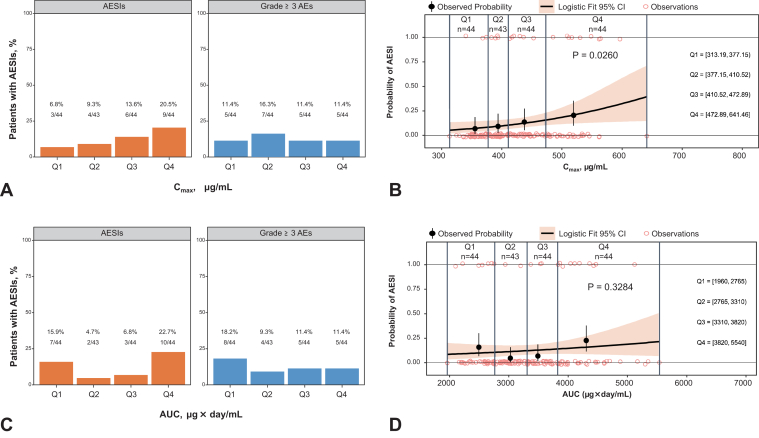
Relationship of frequency of AESIs and all grade greater than or equal to III AEs with quartiles of atezolizumab exposure metric (*A*) C_max_ and (*B*) its probability estimate using logistic regression models and (*C*) AUC and (*D*) its probability estimate using logistic regression models. AE, adverse event; AESI, AE of special interest; AUC, area under the curve; C_max_, maximum plasma concentration; CI, confidence interval; irAE, immune-related adverse event; Q, quartile.

The relationship of atezolizumab exposure with AESIs and all grade greater than or equal to III AEs was estimated using C_max_ and AUC as the exposure metrics. The calculated C_max_ and AUC were evaluated for the frequency of AESIs and all grade greater than or equal to III AEs in each quartile. The results revealed a significant increase in the proportion of patients who developed AESIs in a concentration-dependent manner with increasing quartiles of C_max_ (*p* = 0.0260); no such concentration-dependent changes were observed in the proportion of patients with grade greater than or equal to III AEs (*p* = 0.9316; Fig. 3*A* and *B*). No AUC-dependent changes were observed in the proportion of patients who developed AESIs (*p* = 0.3284) and all grade greater than or equal to III AEs (*p* = 0.2593; Fig. 3*C* and *D*). The details of the grades of AESIs are provided in Supplementary Table 6.

## Discussion

This study evaluated the PK and PopPK of atezolizumab in a subgroup of 175 real-world Japanese patients with unresectable advanced/recurrent NSCLC enrolled in the J-TAIL study.

The atezolizumab plasma concentrations at C2_trough_ were similar to previously reported data from the OAK study.^8^ KM analysis confirmed that OS was significantly shortened when the lowest quartile of C2_trough_ was used as a cutoff, that is, patients with lower C2_trough_ had significantly shortened OS; however, the differences in quartiles of plasma concentration did not affect the objective response rate and PFS. Patient characteristics with an association with C2_trough_ were identified as male sex, high BSA level, nonsquamous cell carcinoma histology, positive smoking history, having adrenal metastases, low Alb level, and high CRP level in the Q1 population compared with the Q2 to Q4 populations. Analysis of the association between atezolizumab-related AEs and exposures calculated using the PopPK model revealed that AUC did not correlate with frequency of AESIs or grade greater than or equal to III AEs, whereas an increased C_max_ significantly increased the frequency of AESIs.

Overall, there are many unanswered questions about the PK of antibody drugs. In general, endogenous IgG has an Fc receptor (FcRn)-mediated recycling mechanism.^22^ Most antibodies incorporated in the endosome escape lysosomal degradation by FcRn-mediated recycling mechanisms, contributing to the increased elimination half-life of IgG. Similar to IgG, serum Alb is also recycled by FcRn,^23^ and it has been reported that between Alb and IgG formulation, the CL of IgG is high when Alb is low, resulting in PK fluctuations.^24^ Furthermore, analysis of the relationship between PK and OS of antibody drugs revealed that the quartile group with the lowest trastuzumab trough PK at the first infusion for gastric cancer had a shorter OS and included patients with an ECOG PS score of greater than or equal to 2 and a higher number of metastatic organs.^25^ We previously reported that the lowest quartile of first-infusion trough PK of ramucirumab for NSCLC was significantly associated with shortened OS, low Alb levels, and high CRP levels.^11^ This study also revealed that patients with low initial PK of atezolizumab had a shorter OS and that this group was characterized by low Alb and high CRP levels. This phenomenon of poor survival in populations with low PK of antibody drugs has been reported repeatedly, suggesting that antibody concentrations are low in patients with impaired conditions, including cancer cachexia, characterized by poor PS, low Alb level, and high CRP level. Cachexia is generally associated with increased protein catabolism in vivo, presumably leading to degradation of antibody drugs with an IgG structure.^26^^,^^27^ Furthermore, Alb level is often decreased in cachexia, which may also lead to increased CL and lower blood levels of antibody drugs. Nevertheless, similar to reports of other antibody drugs, the PK of atezolizumab did not contribute to PFS. This fact indicates that changes in the PK of antibody drugs are not clearly associated with treatment response but rather are prognostic markers as indicators of patients’ compromised status, including cachexia.

The irAEs are characteristic AEs of ICIs and can lead to treatment interruption, discontinuation, or even death. To date, there is no biomarker that can predict the development of irAEs, and treatment with steroids or immunosuppressive drugs may be necessary after the onset of an irAE. In this study, C_max_ and AUC were calculated using the PopPK model, indicating that the frequency of AESIs may increase with increasing C_max_. Previously, Morrissey et al.,^28^ evaluated the exposure–response (ER) relationship using a PopPK model using pooled data from 1228 patients in phase I and III trials of atezolizumab in patients with advanced NSCLC and urothelial carcinoma but found no clear relationship between exposure and efficacy or AEs.

Owing to the small number of patients and analysis points in this study, it is possible that the ER relationship between C_max_ and AESIs was random. Nevertheless, unlike the analysis population in the study by Morrissey et al.^28^ J-TAIL is based on a real-world Japanese patient population that was not enrolled according to the strict criteria of a clinical trial. In general, the frequency of AEs is known to be higher among Japanese patients, and racial differences may also be a factor.^29^ In fact, for pembrolizumab, a 400 mg once every six weeks dosing regimen was recently approved in addition to the 200 mg once every three weeks regimen because the PopPK results revealed increased C_max_ but comparable AUC, although, in fact, an increased incidence of irAEs was reported with the 400 mg once every 6 weeks dosing regimen.^30^ Further investigation of the association between the C_max_ of ICIs and irAEs is needed. This study revealed an association between AESIs and C_max_, but no correlation was found between grade greater than or equal to III AEs and C_max_. IrAEs are caused by abnormal activation of autoimmunity and may occur through a variety of mechanisms, including the production of autoantibodies derived from B cells, cytokine release, and infiltration of normal organ functions by CD8+ T cells. Although the relationship with C_max_ is unclear for AEs evaluated only by grade, we believe that the relationship with C_max_ may become apparent if we focus on specific irAEs. In the future, it will be necessary to understand the pathogenesis of individual irAEs, search for biomarkers accordingly, and determine a course of action.

The limitations of this study include a small, nonrandomized sample size that might affect comparability between groups, variations in sample collection time, and judgment of effectiveness/safety outcomes based on clinicians’ discretion; however, it represents a real-world setting. Nevertheless, given the nature of real-world data, unimputed missing data could potentially negatively affect the interpretation of the results. In this study, PK of atezolizumab was measured in plasma using a proprietary LC–MS assay system. The results of this study were not compared with those of previous enzyme-linked immunosorbent assay methods using serum for atezolizumab PK, which may have resulted in slightly different results.

Previous reports have also revealed that baseline prognostic factors may influence PK and clinical outcomes.^31^, ^32^, ^33^ In addition, other reports have revealed that traditional interpretation of ER analysis is unidirectional and complex time-dependent interactions between response, disease severity, CL, and exposure introduce a bidirectional relationship that makes causality difficult to infer. A disregard for these confounding factors can lead to identification of spurious ER relationships.^34^ This study only conducted external validation and estimated individual PK parameters post hoc for the exposure–response. Furthermore, no definitive conclusions were reached on biomarkers of efficacy/effectiveness and safety. In real-world clinical practice, ICIs are often administered to the elderly, patients with organ dysfunction, and patients with poor PS. Therefore, it is important to conduct real-world studies and use the results to promote reverse translational research in addition to translational research.

In conclusion, the results of this study suggest that in real-world Japanese patients treated with atezolizumab monotherapy for unresectable advanced/recurrent NSCLC, PK was similar to previously reported results, mainly in Western patients. Certain patient populations had shorter OS, and atezolizumab plasma concentrations in cycle 3 were lower in this population. Elevated C_max_ at cycle 1 may be associated with an increased frequency of AESIs. Further research is required to validate the findings of this study.

## CRediT Authorship Contribution Statement

**Shigehiro Yagishita:** Conceptualization, Resources, Investigation, Visualization, Writing—original draft, Writing—review and editing.

**Yasushi Goto:** Conceptualization, Resources, Investigation, Writing—review and editing.

**Makoto Nishio:** Conceptualization, Resources, Supervision, Investigation, Writing—review and editing.

**Hiroaki Akamatsu:** Conceptualization, Resources, Investigation, Writing—review and editing.

**Hidetoshi Hayashi:** Conceptualization, Resources, Investigation, Writing—review and editing.

**Satoru Miura:** Conceptualization, Resources, Investigation, Writing—review and editing.

**Koji Tamada:** Conceptualization, Resources, Investigation, Writing—review and editing.

**Hiroshi Kagamu:** Conceptualization, Resources, Investigation, Writing—review and editing.

**Akinobu Hamada:** Conceptualization, Resources, Investigation, Writing—review and editing.

**Mayu Ohuchi:** Conceptualization, Resources, Investigation, Writing—review and editing.

**Akihiko Gemma:** Conceptualization, Resources, Supervision, Funding acquisition, Investigation, Writing—review and editing.

**Ichiro Yoshino:** Conceptualization, Resources, Investigation, Writing—review and editing.

**Toshihiro Misumi:** Formal analysis, Writing—review and editing.

**Akito Hata:** Resources, Investigation, Writing—review and editing.

**Satoshi Hara:** Resources, Investigation, Writing—review and editing.

**Takashi Kijima:** Resources, Investigation, Writing—review and editing.

**Fujita Masaki:** Resources, Investigation, Writing—review and editing.

**Shunichiro Iwasawa:** Conceptualization, Visualization, Writing—original draft, Project administration, Writing—review and editing.

**Shintaro Nakagawa:** Formal analysis, Writing—review and editing.

**Tetsuya Mitsudomi:** Conceptualization, Resources, Supervision, Funding acquisition, Investigation, Visualization, Project administration, Writing—review and editing.

## Disclosure

Dr. Yagishita has received grants or contracts from Nippon Boehringer Ingelheim and payment or honoraria for lectures, presentations, speaker’s bureau, manuscript writing, or educational events from LSI Medience in the past 36 months. Dr. Goto has received grants or contracts from 10.13039/100004325AstraZeneca and 10.13039/100004319Pfizer; grants for the institution from AbbVie, Eli Lilly, Bristol Myers Squibb, Ono, Novartis, Kyorin, Daiichi Sankyo, Novartis, and Preferred Network; payment or honoraria for lectures, presentations, speaker’s bureau, manuscript writing, or educational events from Eli Lilly, Chugai, Taiho, Boehringer Ingelheim, Ono, Bristol Myers Squibb, Pfizer, Merck Sharp & Dohme, Novartis, Merck, and Thermo Fisher; has participated in data safety monitoring boards or advisory boards for AstraZeneca, Chugai, Boehringer Ingelheim, Eli Lilly, Taiho, Pfizer, Novartis, Guardant Health, Illumina, Daiichi Sankyo, Ono, Bristol Myers Squibb, and Merck Sharp & Dohme; and had a leadership or fiduciary role in Cancer Net Japan and JAMT in the past 36 months. Dr. Nishio has received payment or honoraria for lectures from Ono, Chugai, Taiho, Bristol Myers Squibb, Daiichi Sankyo, Eli Lilly, AstraZeneca, Merck Sharp & Dohme, AbbVie, Takeda, Pfizer, Boehringer Ingelheim, Novartis, Nippon Kayaku, Merck, and Janssen in the past 36 months. Dr. Akamatsu has received grants or contracts from 10.13039/100002429Amgen, Eli Lilly, and 10.13039/100010795Chugai; payment or honoraria for lectures, presentations, speaker’s bureau, manuscript writing, or educational events from Amgen, Ono, AstraZeneca, Pfizer, Boehringer Ingelheim, Takeda, Bristol Myers Squibb, Taiho, Chugai, Eli Lilly, Merck Sharp & Dohme, Nippon Kayaku, and Novartis; and has participated in data safety monitoring boards or advisory boards for Amgen and Janssen in the past 36 months. Dr. Hayashi has received grants or contracts from IQVIA Services, Syneos Health, EPS Corporation, Nippon Kayaku, Takeda, Merck Sharp & Dohme, Amgen, Taiho, Bristol Myers Squibb, Janssen, CMIC, Pfizer, Labcorp Drug Development, Kobayashi, Eisai, EP-CRSU, Shionogi, Otsuka, GlaxoSmithKline, Sanofi, Chugai, Nippon Boehringer Ingelheim, SRL Medisearch, PRA Health Sciences, Astellas Pharma, Ascent Development Services, Bayer Yakuhin, Parexel, Kissei, EPS Corporation, Daiichi Sankyo, Ono, PPD-SNBL, SymBio, Mebix, AstraZeneca, Mochida, Japan Clinical Research Operations, Eli Lilly, Novartis, Otsuka, and SRL; payment or honoraria for lectures, presentations, speaker’s bureau, manuscript writing, or educational events from Ono, Daiichi Sankyo, AstraZeneca, Eli Lilly, Chugai Pharmaceutical, Merck Sharp & Dohme, Pfizer, Nippon Boehringer Ingelheim, Merck, 3H Clinical Trial, Novartis, Bristol Myers Squibb, Amgen, Sysmex Corporation, Takeda, and Guardant Health in the past 36 months. Dr. Miura has received payment or honoraria for lectures from Chugai, Taiho, Ono, AstraZeneca, Bristol Myers Squibb, Eli Lilly, Boehringer Ingelheim, and Takeda in the past 36 months. Dr. Kagamu has received payment or honoraria for lectures from Chugai, Ono, AstraZeneca, and Bristol Myers Squibb in the past 36 months. Dr. Yoshino has received grants or contracts from AstraZeneca, Chugai, Daiichi Sankyo, and Taiho; consulting fees from 10.13039/100004325AstraZeneca, 10.13039/100010795Chugai, 10.13039/501100002736Covidien, and 10.13039/100004331Johnson & Johnson; and payment or honoraria for lectures from AstraZeneca, Chugai, Daiichi Sankyo, Eli Lilly, Covidien, Johnson & Johnson, Taiho, Amgen, Merck Sharp & Dohme, CSL Behring, KM Biologics, Intuitive Surgical, Shionogi, Tsumura, and Takeda in the past 36 months. Dr. Misumi has received honoraria for lectures, presentations, speaker’s bureau, manuscript writing, or educational events from Chugai, AstraZeneca, and Miyarisan in the past 36 months. Dr. Hata has received grants or contracts from Merck Sharp & Dohme, Taiho, Eli Lilly, Chugai, Boehringer Ingelheim, and AstraZeneca, and payment or honoraria for lectures, presentations, speaker’s bureau, manuscript writing, or educational events from Eli Lilly, Merck Sharp & Dohme, Chugai, Pfizer, AstraZeneca, Boehringer Ingelheim, and Taiho in the past 36 months. Dr. Kijima has received grants or contracts from Chugai and payment or honoraria for lectures, presentations, speaker’s bureau, manuscript writing, or educational events from Chugai in the past 36 months. Dr. Mitsudomi has received grants or contracts from Merck Sharp & Dohme, Ono, AstraZeneca, and Chugai; payment or honoraria for speaker’s bureau from Merck Sharp & Dohme, Ono, Bristol Myers Squibb, AstraZeneca, and Chugai; has participated in advisory boards for Merck Sharp & Dohme, Ono, Bristol Myers Squibb, AstraZeneca, and Chugai; and has been a President at IASLC in the past 36 months. The remaining authors declare no conflict of interest.
