# Supplementary figures and images for: Real-World Pharmacokinetics, Effectiveness, and Safety of Atezolizumab in Patients With Unresectable Advanced or Recurrent NSCLC: An Exploratory Study of J-TAIL
Source: JTO Clin Res Rep. 2024 May 16;5(7):100683. doi: 10.1016/j.jtocrr.2024.100683 (PMC11293501; doi:10.1016/j.jtocrr.2024.100683)

## Supplemental material

**Supplemental Figure 1.** Patient disposition

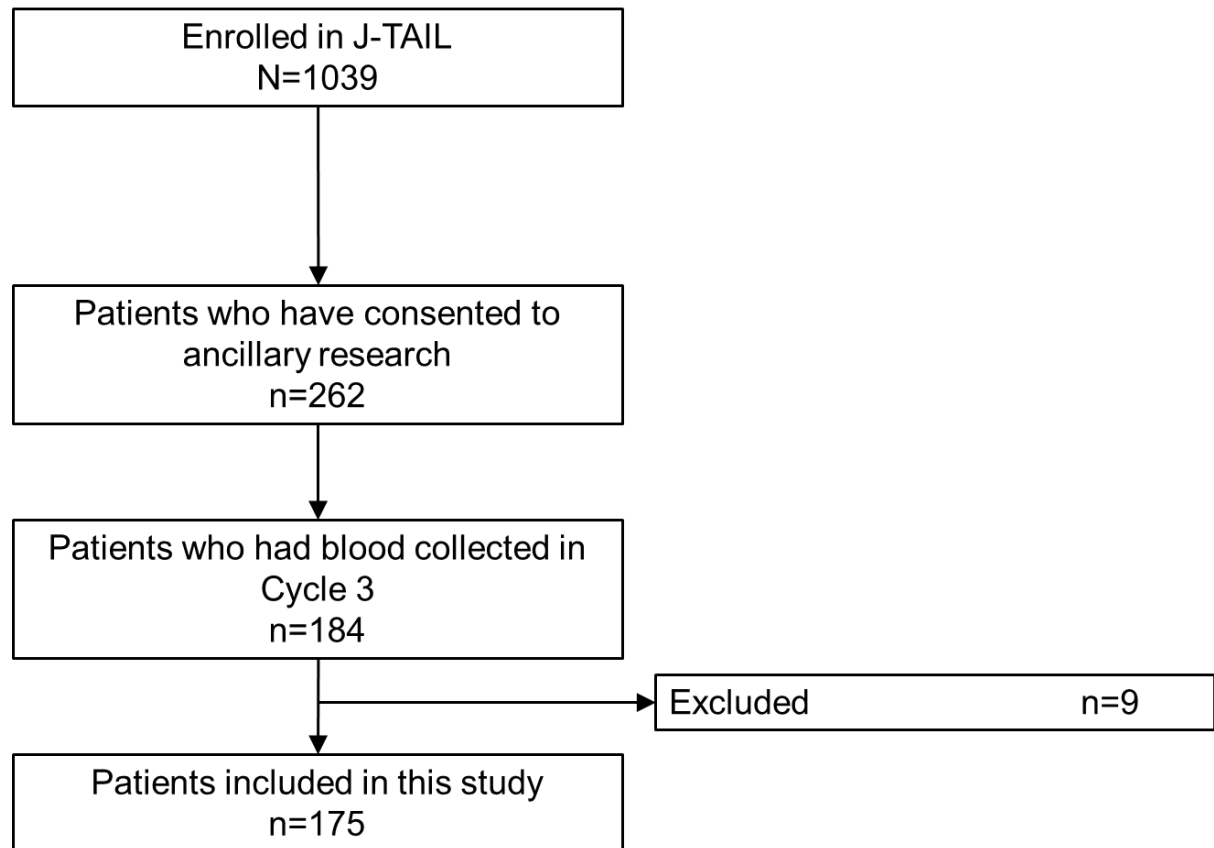

Supplement: Supplemental Figure 1 [file mmc1.pdf]
