# Supplementary material for: Real-World Pharmacokinetics, Effectiveness, and Safety of Atezolizumab in Patients With Unresectable Advanced or Recurrent NSCLC: An Exploratory Study of J-TAIL
Source: JTO Clin Res Rep. 2024 May 16;5(7):100683. doi: 10.1016/j.jtocrr.2024.100683 (PMC11293501; doi:10.1016/j.jtocrr.2024.100683)
Supplement: Supplemental Figure 2 [file mmc2.pdf]

Supplemental Figure 2. Scatter plot of atezolizumab plasma concentrations in quartiles Q1–Q4 in Cycle 3

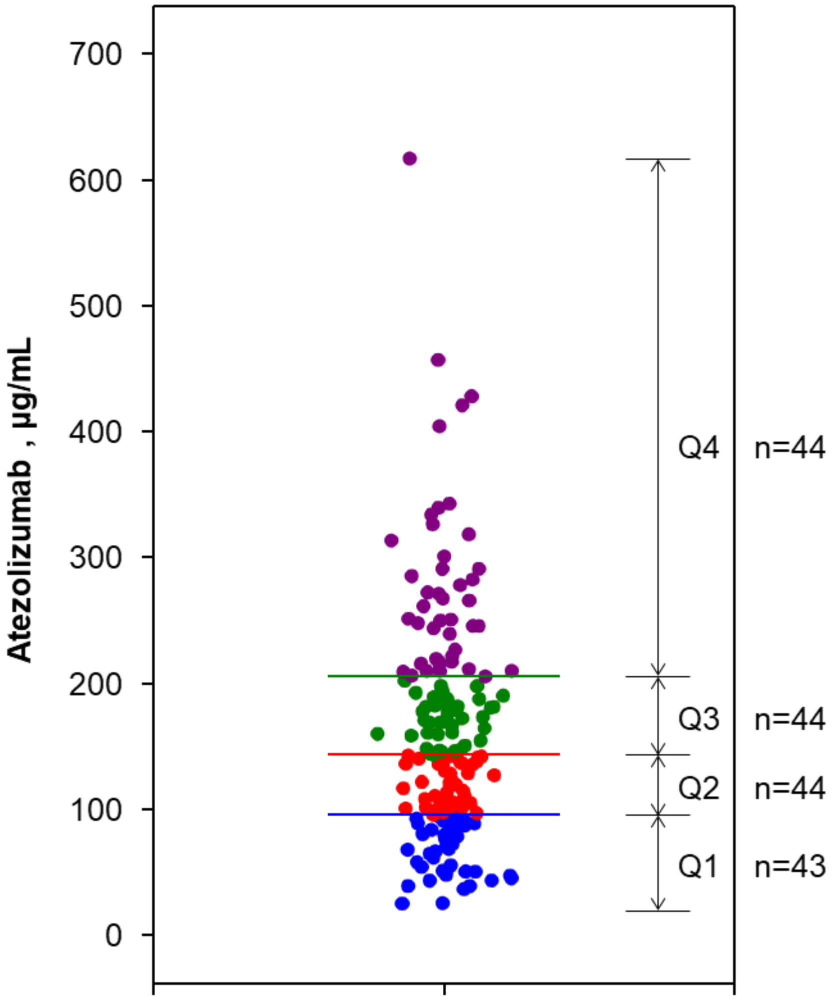

N	175
Mean±SD	160.89±90.03
Median	143.87
Q1, Q3	95.90, 206.20
Min, Max	25.82, 616.58

Max, maximum; min; minimum; Q, quartile; SD, standard deviation.
