# Supplementary material for: Real-World Pharmacokinetics, Effectiveness, and Safety of Atezolizumab in Patients With Unresectable Advanced or Recurrent NSCLC: An Exploratory Study of J-TAIL
Source: JTO Clin Res Rep. 2024 May 16;5(7):100683. doi: 10.1016/j.jtocrr.2024.100683 (PMC11293501; doi:10.1016/j.jtocrr.2024.100683)
Supplement: Supplemental Figure 3 [file mmc3.pdf]

Supplemental Figure 3. KM analysis of (A) PFS compared between Q1 vs Q2–Q4 as individual groups and (B) OS compared between Q1 vs Q2–Q4 as individual groups

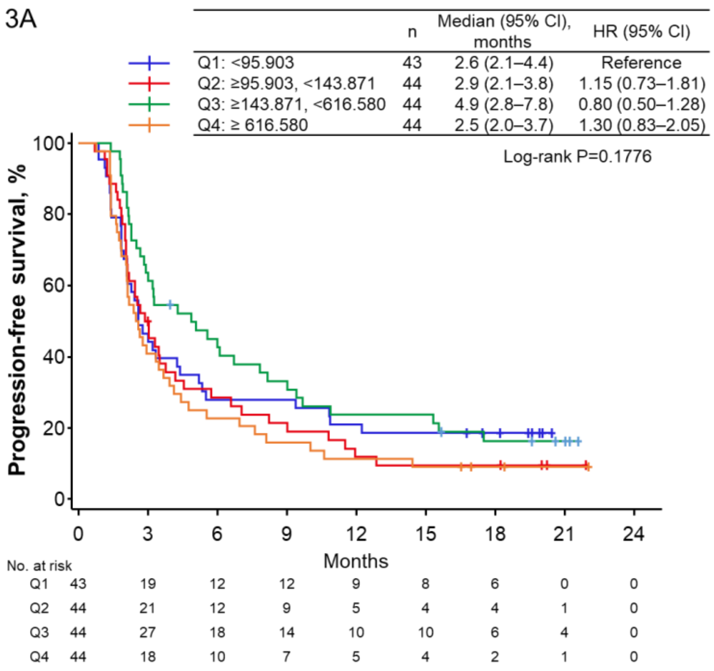

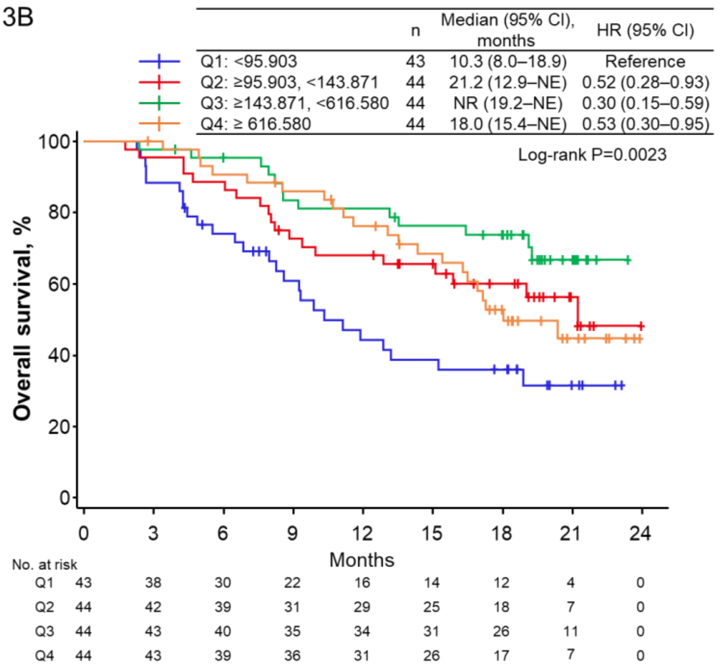

CI, confidence interval; HR, hazard ratio; KM, Kaplan-Meier; NE, not evaluable; NR, not reached; OS, overall survival; PFS, progression-free survival; Q, quartile.
