# Supplementary material for: Real-World Pharmacokinetics, Effectiveness, and Safety of Atezolizumab in Patients With Unresectable Advanced or Recurrent NSCLC: An Exploratory Study of J-TAIL
Source: JTO Clin Res Rep. 2024 May 16;5(7):100683. doi: 10.1016/j.jtocrr.2024.100683 (PMC11293501; doi:10.1016/j.jtocrr.2024.100683)
Supplement: Supplemental Table 1 [file mmc4.pdf]

Supplemental Table 1. Atezolizumab and rituximab digested and extracted to obtain signature peptides

Drug	Selected peptide	Optimal MRM condition			
		Transition mass filter [m/z]	Q1 [V]	Collision [V]	Q3 [V]
Atezolizumab	RHWPGGFDYWGQGTLVTVSSASTK	660.25 > 880.20	−32	−14	−40
Rituximab (IS for atezolizumab)	GLEWIGAIYPGNGDTSYNQK	1092.10 > 1180.40	−36	−35	−44

IS, internal standard; MRM, multiple reaction monitoring; m/z, mass-to-charge ratio; Q, quartile.
