# Supplementary material for: Real-World Pharmacokinetics, Effectiveness, and Safety of Atezolizumab in Patients With Unresectable Advanced or Recurrent NSCLC: An Exploratory Study of J-TAIL
Source: JTO Clin Res Rep. 2024 May 16;5(7):100683. doi: 10.1016/j.jtocrr.2024.100683 (PMC11293501; doi:10.1016/j.jtocrr.2024.100683)
Supplement: Supplemental Table 2 [file mmc5.pdf]

Supplemental Table 2. Intraday and interday stability of atezolizumab

		Intra−assay (1 day, n=5)			Inter−assay (3 days, n=15)		
**Drug**	**Concentration (µg/mL)**	**Measured concentration (µg/mL) (mean ± SD)**	**Accuracy (%)**	**CV (%)**	**Measured concentration (µg/mL) (mean ± SD)**	**Accuracy (%)**	**CV (%)**
Atezolizumab	5	4.66 ± 0.85	93.2	18.2	5.52 ± 0.87	110.4	15.8
	15	14.5 ± 2.07	96.7	14.3	15.1 ± 1.57	100.6	10.4
	75	79.2 ± 6.12	105.6	7.73	73.1 ± 7.44	97.4	10.2
	480	452.2 ± 32.7	94.2	7.23	456.7 ± 31.2	95.2	6.8

CV, coefficient of variation; SD, standard deviation.
