# Supplementary material for: Real-World Pharmacokinetics, Effectiveness, and Safety of Atezolizumab in Patients With Unresectable Advanced or Recurrent NSCLC: An Exploratory Study of J-TAIL
Source: JTO Clin Res Rep. 2024 May 16;5(7):100683. doi: 10.1016/j.jtocrr.2024.100683 (PMC11293501; doi:10.1016/j.jtocrr.2024.100683)
Supplement: Supplemental Table 3 [file mmc6.pdf]

Supplemental Table 3. Short-term stability of atezolizumab

Parameters for stability studies	Atezolizumab			
	15.0 µg/mL		480 µg/mL	
	Measured concentration (µg/mL) (mean ± SD)	Accuracy (%)	Measured concentration (µg/mL) (mean ± SD)	Accuracy (%)
Short-term stability in plasma for 6 h at room temperature (n=3)	14.3 ± 1.45	95.5	490.6 ± 11.9	102.2
Stability in plasma during freeze (−80°C) and thaw cycles in Cycle 3 (n=5)	14.5 ± 1.80	96.6	472.6 ± 19.3	98.5
Long-term stability in plasma for 30 days at −80°C (n=3)	14.0 ± 2.07	93.4	447.0 ± 37.3	93.1
Processed sample stability in HPLC set at 5°C for 24 h (n=5)	16.8 ± 1.6	112.1	493.4 ± 51.0	102.8

HPLC, high-performance liquid chromatography; SD, standard deviation.
