# Supplementary material for: Real-World Pharmacokinetics, Effectiveness, and Safety of Atezolizumab in Patients With Unresectable Advanced or Recurrent NSCLC: An Exploratory Study of J-TAIL
Source: JTO Clin Res Rep. 2024 May 16;5(7):100683. doi: 10.1016/j.jtocrr.2024.100683 (PMC11293501; doi:10.1016/j.jtocrr.2024.100683)
Supplement: Supplemental Table 4 [file mmc7.pdf]

Supplemental Table 4. Calibration curve for atezolizumab

	**Atezolizumab**		
	**Day 1**	**Day 2**	**Day 3**
a	0.0186	0.0163	0.0158
b	0.0209	−0.0230	−0.0204
R^2	0.9868	0.9950	0.9959
r	0.9934	0.9975	0.9979

The approximate equation for the calibration curve was y=ax+b, and the correlation coefficients R^2 and r were calculated.
