# Supplementary material for: Real-World Pharmacokinetics, Effectiveness, and Safety of Atezolizumab in Patients With Unresectable Advanced or Recurrent NSCLC: An Exploratory Study of J-TAIL
Source: JTO Clin Res Rep. 2024 May 16;5(7):100683. doi: 10.1016/j.jtocrr.2024.100683 (PMC11293501; doi:10.1016/j.jtocrr.2024.100683)
Supplement: Supplemental Table 5 [file mmc8.pdf]

Supplemental Table 5. Summary statistics of exposure parameters of atezolizumab

Parameter		J-TAIL<br>N=175
**AUC Cycle 1 (µg·day/mL)**	Mean (SD)	3330 (722)
	Median [Min, Max]	3310 [1960, 5540]
	Geo. mean (Geo. CV%)	3260 (21.7)
**C_{max} Cycle 1 (µg/mL)**	Mean (SD)	427 (64.8)
	Median [Min, Max]	411 [313, 641]
	Geo. mean (Geo. CV%)	422 (14.8)
**C_{min} Cycle 1 (µg/mL)**	Mean (SD)	97.9 (33.6)
	Median [Min, Max]	95.7 [34.6, 214]
	Geo. mean (Geo. CV%)	92.2 (36.9)
**C_{min} Cycle 2 (µg/mL)**	Mean (SD)	156 (60)
	Median [Min, Max]	150 [45.1, 372]
	Geo. mean (Geo. CV%)	144 (42.7)

AUC, area under the curve; C_{max}, maximum plasma concentration; C_{min}, minimum plasma concentration; CV%, coefficient of variation; Geo., geometric; max, maximum; min, minimum; SD, standard deviation.
