# Supplementary material for: Real-World Pharmacokinetics, Effectiveness, and Safety of Atezolizumab in Patients With Unresectable Advanced or Recurrent NSCLC: An Exploratory Study of J-TAIL
Source: JTO Clin Res Rep. 2024 May 16;5(7):100683. doi: 10.1016/j.jtocrr.2024.100683 (PMC11293501; doi:10.1016/j.jtocrr.2024.100683)
Supplement: Supplemental Table 6 [file mmc9.pdf]

Supplemental Table 6. Details of AESIs

AESI	Grade 1	Grade 2	Grade 3	Grade 4	Grade 5
Total	7 (4.0)	9 (5.1)	4 (2.3)	0 (0.0)	2 (1.1)
Diarrhea	4 (2.3)	0 (0.0)	0 (0.0)	0 (0.0)	0 (0.0)
Interstitial lung disease	1 (0.6)	1 (0.6)	0 (0.0)	0 (0.0)	1 (0.6)
Hypothyroidism	0 (0.0)	3 (1.7)	0 (0.0)	0 (0.0)	0 (0.0)
Colitis	1 (0.6)	1 (0.6)	1 (0.6)	0 (0.0)	0 (0.0)
Adrenal insufficiency	0 (0.0)	2 (1.1)	1 (0.6)	0 (0.0)	0 (0.0)
Liver dysfunction	0 (0.0)	1 (0.6)	1 (0.6)	0 (0.0)	0 (0.0)
Myocarditis	1 (0.6)	0 (0.0)	0 (0.0)	0 (0.0)	0 (0.0)
Secondary adrenocortical insufficiency	0 (0.0)	0 (0.0)	1 (0.6)	0 (0.0)	0 (0.0)
Injection-related reactions	0 (0.0)	1 (0.6)	0 (0.0)	0 (0.0)	0 (0.0)
Pulmonary inflammation	0 (0.0)	0 (0.0)	0 (0.0)	0 (0.0)	1 (0.6)

AESI, adverse event of special interest.
